# Study about Food Choice Determinants According to Six Types of Conditioning Motivations in a Sample of 11,960 Participants

**DOI:** 10.3390/foods9070888

**Published:** 2020-07-07

**Authors:** Raquel P. F. Guiné, Elena Bartkiene, Viktória Szűcs, Monica Tarcea, Marija Ljubičić, Maša Černelič-Bizjak, Kathy Isoldi, Ayman EL-Kenawy, Vanessa Ferreira, Evita Straumite, Małgorzata Korzeniowska, Elena Vittadini, Marcela Leal, Lucia Frez-Muñoz, Maria Papageorgiou, Ilija Djekić, Manuela Ferreira, Paula Correia, Ana Paula Cardoso, João Duarte

**Affiliations:** 1 CERNAS Research Centre, Polytechnic Institute of Viseu, 3504-510 Viseu, Portugal; paulacorreia@esav.ipv.pt; 2 Department of Food Safety and Quality, Lithuanian University of Health Sciences, 44307 Kaunas, Lithuania; elena.bartkiene@lsmuni.lt; 3 Directorate of Food Industry, Hungarian Chamber of Agriculture, H119 Budapest, Hungary; szucs.viktoria@nak.hu; 4 Department of Community Nutrition & Food Safety, University of Medicine, Pharmacy, Science and Technology, 540139 Targu-Mures, Romania; monica.tarcea@umfst.ro; 5 Department of Pediatrics, General Hospital Zadar, 23000 Zadar, Croatia; marija.ljubicic@zd.t-com.hr; 6 Faculty of Health Sciences, University of Primorska, 6310 Izola, Slovenia; Masa.Cernelic@fvz.upr.si; 7 Department of Biomedical, Health and Nutrition Sciences, Long Island University, 720 Northern Boulevard, Brookville, New York, NY 11548-1327, USA; kathy.isoldi@liu.edu; 8 Department of Molecular Biology, Genetic Engineering and Biotechnology Institute, University of Sadat City, Sadat City 79/22857, Egypt; elkenawyay@yahoo.com; 9 Department of Nutrition, School of Nursing, UFMG University, Belo Horizonte 30130-100, Brazil; vanessa.nutr@gmail.com; 10 Department of Food Technology, Latvia University of Life Sciences and Technologies, LV 3001 Jelgava, Latvia; evita.straumite@llu.lv; 11 Faculty of Food Science, Wrocław University of Environmental and Life Sciences, 51-630 Wrocław, Poland; malgorzata.korzeniowska@upwr.edu.pl; 12 School of Biosciences and Veterinary Medicine, University of Camerino, 62032 Camerino, Italy; elena.vittadini@unicam.it; 13 School of Nutrition, Faculty of Health Sciences, Maimonides University, Buenos Aires C1405, Argentina; leal.nutricion@gmail.com; 14 Food Quality and Design Group, Wageningen University & Research, 6700 HB Wageningen, The Netherlands; lucia.frezmunoz@wur.nl; 15 Alexander Technological Educational Institute, Department Food Technology, 57400 Thessaloniki, Thessaloniki, Greece; mariapapage@food.teithe.gr; 16 Faculty of Agriculture, University of Belgrade, 11000 Belgrade, Serbia; idjekic@agrif.bg.ac.rs; 17 UICISA:E Research Centre, Polytechnic Institute of Viseu, 3504-510 Viseu, Portugal; mmcferreira@gmail.com (M.F.); duarte.johnny@gmail.com (J.D.); 18 CIDEI Research Centre, Polytechnic Institute of Viseu, 3504-510 Viseu, Portugal; a.p.cardoso@esev.ipv.pt

**Keywords:** eating determinants, healthy diet, emotions, feeding behavior, socio-cultural environment, instrument validation

## Abstract

Many aspects linked to personal characteristics, society and culture constitute some of the motivators that drive food choice. The aim of this work was to determine in what extent the eating behaviors of individuals are shaped by six different types of determinants, namely: health, emotions, price and availability, society and culture, environment and politics, and marketing and commercials. This is a descriptive cross-sectional study, involving a non-probabilistic sample of 11,960 participants from 16 countries. The objective of this work was to validate the questionnaire, so as to make it suitable for application in different contexts and different countries. For that, six scales were considered for validation by confirmatory factor analysis with structural equation modelling. The obtained results showed that the six individual scales evaluated presented good or very good fitting indices, with saturation in goodness-of-fit index in all cases. The values of chi-square ratio were 6.921 (for health), 0.987 (environment), 0.610 (emotions) and 0.000 in the remaining cases (convenience, society, marketing). Furthermore, the fit was perfect, with saturation for all indices, in three of the six models (convenience, society and marketing). The results of this wok allowed the validation of the six scales, and the assessing of different types of factors that can influence food choices and eating behaviors, namely in the categories: health, emotions, price and availability, society and culture, environment and politics, and marketing and commercials.

## 1. Introduction

Dietary patterns depend on everyday food choices, and include aspects like quantity, proportion, variety and combinations or frequencies of consumption. Knowledge about food choices and which factors may determine what people select to consume are important from the social, as well as the health, point of view. The aspects linked to society and culture are some of the motivators that drive food choice [1,2].

The food environment in western societies has been recognized as tending too much and too fast to the unhealthy side of eating, being designated by some as “toxic” [3]. People are constantly exposed to unhealthy food in supermarkets, food shops, restaurants (most especially fast food) or vending machines. This constant appeal of unhealthy food is well known to contribute to unhealthy food choices, which lead to epidemic burdens of obesity, diabetes, heart diseases and other chronic diseases [3,4,5]. In the opposite trend are the consumers who value their health and food as health-enhancing motors. These individuals tend to make appropriate and careful food choices, opting for a more balanced diet and favoring the consumption of functional foods. It is known that the market of functional foods and nutraceuticals has experienced a very fast growing rate in the past few decades [5]. In this way, health and disease can act as motivators to influence people’s food choices [3,4,5,6,7,8].

Emotions are fundamental in all aspects of life, and eating is not an exception. People tend to be conditioned to some extent in their eating behaviors according to their emotional patterns, or even to make variable food choices according to a momentary mood. Emotional eating corresponds to a tendency to overeat as a response to negative emotions such as anxiety or irritability [9]. On the other hand, sadness, loneliness or depression can impede many people from eating the foods necessary for the correct functioning of their body [10]. Hence the role of emotions in determining food choices is incredibly relevant [9,10].

Furthermore, economic factors have a very marked influence on eating habits, and in countries with a low household income the differences are marked, as compared with the more industrialized, more urbanized and more globalized societies, which undergo a nutritional transition. Besides income, availability issues also contribute to shaping eating behaviors, most especially in today’s globalized markets, with goods being traded between many different countries [1,11].

The role of consumption and consumer behavior is increasingly considered in the food supply chain. Sustainable consumption or green consumer behavior corresponds to customers’ choices and refusals to buy and consume products harmful to the environment, and as alternatives seek to purchase products that have a minimal impact on the environment, or preferably, that are beneficial for the global sustainability. Because sustainable consumption behaviors can significantly diminish the social and environmental impacts, more and more people are taking these aspects into account when making their food choices [12,13,14].

The development of a valid and reliable instrument for assessing the factors taken into consideration by consumers when making their food choices is not only a matter of purely academic interest, but it also impacts many different aspects of society, namely health, economy, society, environment, just to name a few. The use of a validated instrument allows for reliability in the data gathered, and guarantees its applicability on a broader scale, particularly when that validation is carried out with data obtained from different sources, like for example different countries [15,16,17,18].

Because the factors that may determine the eating behaviors of individuals can be of very differentiated natures, and have variable degrees of influence on people’s food choices, this work intended to test six different complementing scales for eating motivations, namely: health, emotions, price and availability, society and culture, environment and politics, and marketing and commercials. As such, the objective was to validate the questionnaire and its six scales, so as to make them suitable for application in different contexts and different countries. The validation process followed was a confirmatory factor analysis with structural equation modelling.

## 2. Materials and Methods

### 2.1. Questionnaire

The instrument used for this research was developed by Ferrão et al. [18] with the purpose of addressing different groups of food motivations. The EATMOT project uses a questionnaire that was developed to explore in what way some personal, psychological and social motivations can influence food choices and eating practices. The questionnaire was prepared and previously validated for a study carried out only in Portugal [18], and then it was translated into the native languages of the 15 participating countries, following a back-translation methodology for validation. For the translation process, all the issues related to the possible cultural influences in the interpretation of the questions were verified. The questionnaire structure included different sections, intended to collect information believed relevant for the study, specifically accounting for groups of questions related to six different types of food motivations: Section 1—Health motivations, Section 2—Emotional motivations, Section 3—Economic and Availability motivations, Section 4—Social and Cultural motivations, Section 5—Environmental and Political motivations, Section 6—Marketing and Commercials motivations. All questions in these six sections of the questionnaire are presented in detail in Appendix A.

The participants would express their level of agreement with each statement on the following 5 points hedonic scale: 1—strongly disagree, 2—disagree, 3—neither agree nor disagree, 4—agree and 5—strongly agree. Because some of the questions were in the inverted mode (Q1.5, Q1.9, Q6.1, and Q6.4), the corresponding scores were reversed. In this way, the higher the global scores, the stronger the influence on the food choice and eating processes.

### 2.2. Data Collection

The questionnaire was applied to adult participants, over 18 years old, who answered it voluntarily, anonymously, and after informed consent. All ethical procedures were strictly followed when designing and applying the questionnaire, and it was ensured that the data provided was kept strictly confidential, i.e., no individual responses could ever be associated with the respondent. The survey was approved by the Ethical Committee of Polytechnic Institute of Viseu, with reference nº 04/2017, and follows national and international protocols for research on humans. The sample was selected by convenience and consisted of 11,960 individuals aged between 18 and 90 years, from which the majority were female (71%). The participants were from 16 countries situated on three continents (Europe, America and Africa), and were distributed as: Argentina (4%), Brazil (6%), Croatia (13%), Egypt (7%), Greece (4%), Hungary (4%), Italy (5%), Latvia (5%), Lithuania (4%), Netherlands (4%), Poland (5%), Portugal (11%), Serbia (4%), Slovenia (9%), Romania (7%) and United States of America (7%).

### 2.3. Analysis of the Data

The reliability studies to evaluate the internal consistency were made by means of the Pearson’s linear correlation coefficient (r) and the Cronbach’s alpha. The values of alpha reported by Marôco [19] were used as references: > 0.9 excellent; 0.8–0.9 very good; 0.7–0.8 good; 0.6–0.7 medium; 0.5–0.6 reasonable; < 0.5 bad. However, the same author admits that in the social sciences the adoption of alpha values above 0.5 is plausible. A good definition of the factor implies that items with correlations to the overall score lower than 0.2 when it contains this particular item should not be considered [20,21,22].

For each scale, the factorial solution that emerged through confirmatory factorial analysis (CFA) was tested using the AMOS 24 software (Analysis of Moment Structures). The covariance matrix and the maximum likelihood estimation (MLE) algorithm for parameter estimation were considered [23]. The latent variables (exogenous and/or endogenous) are represented by larger circles and the indicators (measures observed) by rectangles, while the errors are represented by small circles. The following parameters were considered for evaluation: (i) factorial weights; (ii) variances and covariates of the individual reliability of the indicators; (iii) variances and covariances of the factors; and (iv) error correlations [24]. Model acceptance was decided according to: (i) the interpretability, (ii) the modification indexes proposed by the AMOS, and (iii) the model adjustment indicators [24].

Regarding the interpretation of the parameters, the reference values considered were: correlation between the factors (Φ)—the higher the coefficients, the better; regression coefficients (λ)—values greater than 0.50; individual reliability of indicators (δ)—coefficients equal to or greater than 0.25; statistical significance—*p*-value lower than 0.05 [19].

For the indicators of the quality of adjustment of the model, the following reference values were adopted [25,26]:

(a) Values used for absolute fit: Ratio of chi-square and degrees of freedom (χ^2^/df)—if (χ^2^/df) is equal to 1 the fit is perfect, for values lower than 2 it is good, for values lower than 5 it is acceptable and for values greater than 5 is unacceptable. Root mean square residual (RMR)—the lower the value of RMR the better is the fit, so RMR = 0 indicates a perfect fit. Standardized root mean square residual (SRMR)—a value of zero indicates a perfect fit and values lower than 0.08 are generally considered a good fit. Goodness of fit index (GFI)—values around 0.95 or higher are recommended (with maximum value equal to 1), but values over 0.90 are considered a good fit.
(b) Values for relative fit: Comparative fit index (CFI), which is an additional comparative index of the adjustment to the model—values lower than 0.90 indicate a poor fit, values between 0.90 and 0.95 indicate a good adjustment and above 0.95 a very good adjustment (maximum value of 1 corresponds to perfect fit). This index is independent of the sample size.
(c) Population discrepancy index: Root mean square error of approximation (RMSEA)—reference values for the RMSEA, with a 90% confidence interval, between 0.05 and 0.08 mean the adjustment is good, while it is considered very good when the index is lower than 0.05.

## 3. Results

### 3.1. Health Motivations

Structural Equation Modelling has been used by different researchers, such as Guiné et al. [17], for the development of a scale to measure knowledge about dietary fiber, or by Sidali et al. [27], to assess the acceptance of insect-based food coming from the Ecuadorian Amazon rainforest by western students. SEM was also used by Lagerkvist et al. [28] to estimate a construct that could explain consumer confidence in food safety practices along the food supply chain, and Lim et al. [29] used SEM to assess the relationship between food safety knowledge, attitude and behavior among household food preparers. Ting et al. [30] used SEM to model tourists’ food consumption intentions at their destination.

Table 1 shows the statistics (mean and standard deviation) and the correlations of each item with the global value. Analyzing the average indices of the items and corresponding standard deviations, it was found that they are well cantered, since all the items have observed average indices higher than the central score.

**Figure 1:**
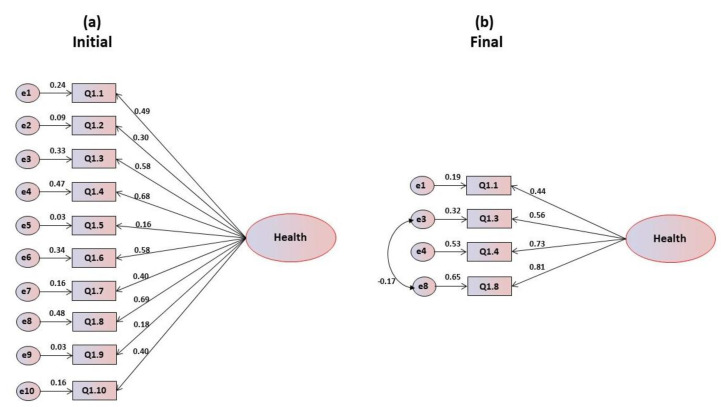
(**a**) Initial and (**b**) final models for the health motivations’ scale.

Table 1 also shows that the item-total correlation coefficients (r) indicate that item Q1.5 is the most problematic, with r = 0.221s and the maximum correlation is obtained in item Q1.6 (r = 0.509), which accounts for about 36% of its variability. Regarding the values of Cronbach’s alpha, these are classified as medium to good since they range from 0.663 in item Q1.6s to 0.713 in item Q1.5, with a global alpha that is also good (α = 0.709). Confirmatory factorial analysis showed that the coefficients of asymmetry and kurtosis presented normal values, oscillating for asymmetry in absolute values between 0.146 and 0.735, and for kurtosis between 0.057 and 0.877, with a Mardia’s coefficient of 0.283 for multivariate normality test. The critical ratios are significant, but from Figure 1a it can be observed that items Q1.2, Q1.5 and Q1.9 have saturations lower than 0.40, which was the lower limit considered as recommended by Marôco [26] for the original studies, and therefore they were eliminated from the model. The same type of analysis led successively to the elimination of other items that presented problems of multicollinearity, resulting in the final refined model represented in Figure 1b. Table 1 also presents the global adjustment indices of the one-dimensional model for health motivations. In the first model, all indices revealed poor or inadequate values, but after refinement the indices presented very good values with saturated index for GFI.

### 3.2. Emotional Motivations

**Figure 2:**
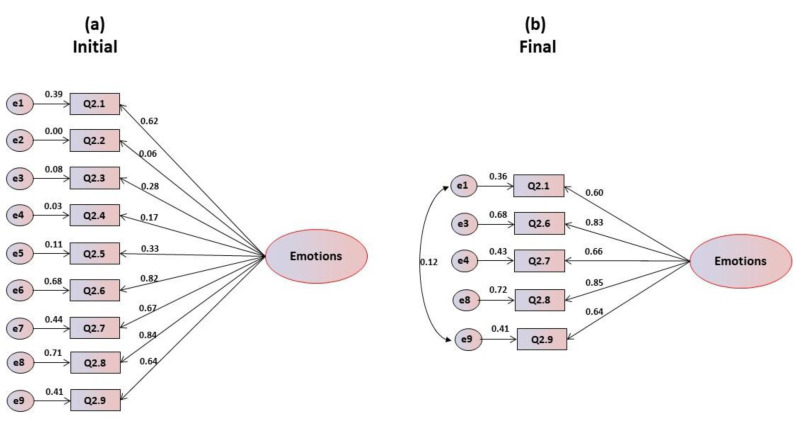
(**a**) Initial and (**b**) final models for the emotional motivations’ scale.

The statistics (mean and standard deviation) and the correlations of each item with the global value are presented in the Table 2. An analysis of the average scores and standard deviations of the items indicates that some items are in the threshold of the central position, which may turn out problematic for the consistency of the scale, since the trend of responses is focused more on the neutral position. The correlation coefficient’s (r) item-total shows that item Q2.2 (r = 0.108) should be excluded because it is lower than the reference value (0.20). The maximum correlation is obtained for item Q2.8 (r = 0.669), which explains about 59% of its variability. The values of Cronbach’s alpha can be classified as medium to good, as they range from 0.689 in item Q2.8, to 0.774 in item Q2.2, with a value for the overall alpha (α = 0.772) that is considered good. When the unifactorial model was submitted to confirmatory factorial analysis, it was shown that the coefficients of skewness and kurtosis presented absolute values corresponding to normality, ranging from 0.044 to 0.657 for skewness and between 0.241 and 1.158 for kurtosis, with a multivariate Mardia’s coefficient of 0.257. The critical ratios are significant, which could lead to the maintenance of all items. However, as indicated in Figure 2a, items Q2.2, Q2.3, Q2.4 and Q2.5 have factorial weights below 0.40, which is the lowest limit recommended by Marôco [26] for original studies, and therefore they have been eliminated, thus giving the final model illustrated in Figure 2b. This final model resulted from the elimination of the aforementioned items plus the refinement through the modification indices proposed by AMOS. It can be observed that in the final model all items have saturations greater than 0.50, and the goodness of fit indices for the overall adjustment can be classified as very good according to Table 2, with GFI and CFI values of 1, which corresponds to perfect fit, and a value of RMSEA of zero, also indicative of a perfect fit.

### 3.3. Economic and Availability Motivations

**Figure 3:**
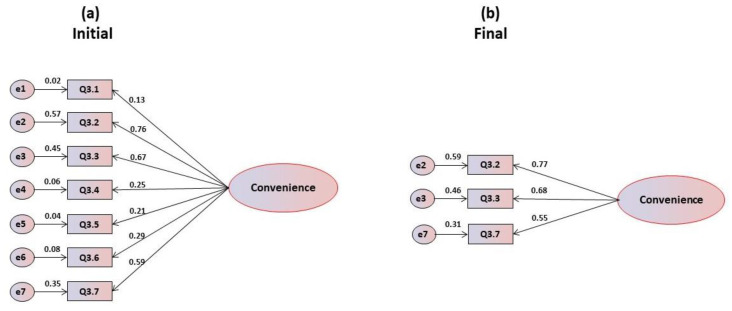
(**a**) Initial and (**b**) final models for the economic and availability motivations’ scale.

By analysis of the mean scores and standard deviations of the items in the scale for economic and availability motivations (Table 3), it is pointed out that only item Q3.7 is below the central threshold, being the most problematic item. Regarding the corrected item-total coefficients, it is observed that items Q3.1, Q3.4 and Q3.7 present correlations lower than 0.20, and therefore, in a more incisive analysis, they should be eliminated. The maximum correlation is obtained for item Q3.6 (r = 0.411), which accounts for 22.6% of its variability. Cronbach’s alpha values range from bad to reasonable, varying from 0.383 in item Q3.2 to 0.582 in item Q3.4, also with an acceptable overall alpha of (α = 0.500). Again, the unifactorial structure was submitted to confirmatory factorial analysis. Analyzing the statistics regarding the normality of the items, it was observed that they all presented absolute values for both asymmetry and kurtosis within the reference values, which were respectively lower than 3.0 and 7.0 for skewness and kurtosis. The multivariate coefficient of Márdia is 0.257, and the critical ratios resulting from the trajectories of the items to the factor are statistically significant. Figure 3a illustrates the initial model, allowing us to verify that items Q3.1, Q3.4, Q3.5 and Q3.6 present saturations lower than 0.40, leading to their elimination. Further, the goodness of fit indices of global adjustment for this model are inadequate (Table 3). By eliminating the items and refining the model, only 3 items remain in the final model because they present saturations higher than the minimum recommended (Figure 3b). For this final model, the goodness of fit indices of global adjustment are all saturated: CFI and GFI are equal to 1, and the remaining values are all equal to zero (Table 3).

### 3.4. Social and Cultural Motivations

Regarding items of the social and cultural motivations’ scale, as it can be observed in Table 4, the corrected correlation coefficient’s item-total shows that items Q4.1, Q4.5 and Q4.8 present correlations lower than 0.20, leading to the recommendation of the elimination of such items. The maximum correlation is obtained for item Q4.4 (r = 0.359), which accounts for 42.6% of its variability. Cronbach’s alpha values oscillate between the inadequate and reasonable, ranging from 0.426 in item Q4.4 to 0.548 in item Q4.5, with the global alpha being acceptable (α = 0.504).

**Figure 4:**
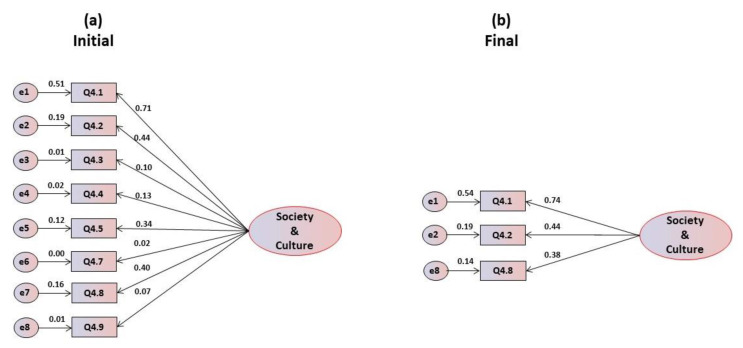
(**a**) Initial and (**b**) final models for the social and cultural motivations’ scale.

The confirmatory factor analysis of the hypothesized unifactorial structure revealed that all the items present a normal distribution, with asymmetry and kurtosis values within the reference values, respectively, lower than 3.0 and 7.0. The multivariate coefficient of Márdia is 0.231. The critical ratios resulting from the trajectories of the items to the factor are statistically significant, which lead to their maintenance. Figure 4a presents the initial model, which excluded item Q4.6 due to multicollinearity problems, and indicates that items Q4.3, Q4.4, Q4.5, Q4.7 and Q4.9 show saturation below 0.40, leading to their elimination. The goodness of fit indices for the global adjustment are inadequate for this initial model (Table 4). When refining the model and eliminating the unsuitable items, only three items remain in the unifactorial structure (Figure 4b). Although one of the items revealed a saturation of 0.38 (under 0.4 but in the limit of acceptance), it was decided to maintain it due to its importance to the structure of the factor. Table 4 also presents the global adjustment indices for this one-dimensional model, and, while in the initial model all indexes were inadequate, in the final model, after refinement, the indexes are excellent, all being saturated.

### 3.5. Environmental and Political Motivations

**Figure 5:**
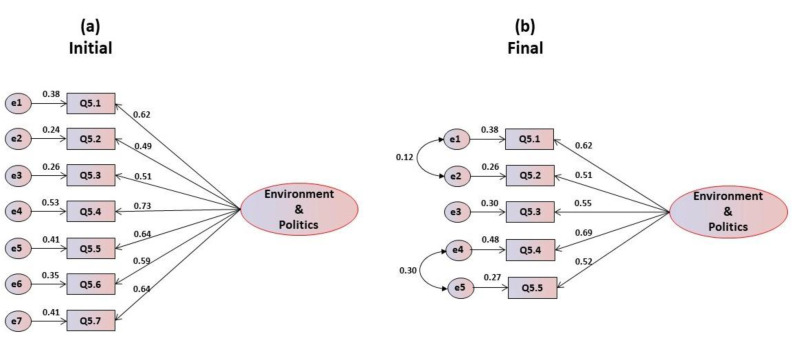
(**a**) Initial and (**b**) final models for the environmental and political motivations’ scale.

Regarding the environmental and political motivations, an analysis of the mean scores and standard deviations of the items (Table 5) shows that they are well centered, being above the central score. The correlation coefficient’s item-total presents values above 0.20, which leads to the maintenance of all items. Cronbach’s alpha values are good for all items, between 0.753 in item Q5.4 and 0.789 in item Q5.2, while the overall alpha is on the threshold of very good (α = 0.799). The confirmatory factorial analysis of the proposed unifactorial structure revealed that all items present a normal distribution, with values of asymmetry and kurtosis within the reference values, oscillating in absolute values for skewness between 0.031 and 0.773, and for kurtosis between 0.054 and 0.599. The multivariate coefficient of Márdia is 0.205. The critical ratios are statistically significant, which leads to the maintenance of all items. Figure 5a represents the initial model, from which it can be observed that all items saturate above 0.40, leading to their maintenance. The indices for evaluation of the goodness of global fit in this first model are adequate, with the exception of the χ^2^/df, which is too high (Table 5). In Figure 5b, the final model is presented after the refinement of the modification indexes proposed by AMOS. It is important to note that, due to multicollinearity problems, items Q5.6 and Q5.77 have been eliminated when defining the final model. The goodness of fit indices for the global adjustment in the final model are very good. While only the chi-square ratio was inadequate for the initial one-dimensional model, in the final model, after refinement, the indexes are excellent, being saturated for GFI, CFI and RMSEA (Table 5).

### 3.6. Marketing and Commercials Motivations

**Figure 6:**
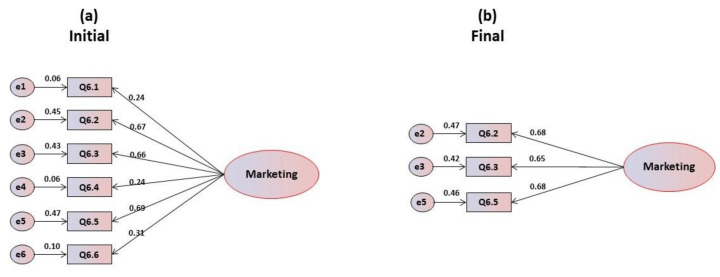
(**a**) Initial and (**b**) final models for the marketing and commercials motivations’ scale.

The mean scores and corresponding standard deviations of the six items in the marketing and commercials’ scale presented in Table 6 show that, in general, they are well cantered, the most problematic item being Q6.2, whose observed value is lower than average. The correlation coefficient’s item-total reveals very weak and negative correlations in items Q6.1 and Q6.4, which leads to the rejection of these items in a more refined analysis. Cronbach’s alpha values are inadmissible, with an overall alpha value of 0.399. However, it was still decided to perform a confirmatory factor analysis in order to determine the possible performances of the items. The confirmatory factorial analysis carried out on the unifactorial structure revealed that all items present a normal distribution, with values of skewness and kurtosis within the reference values. The multivariate coefficient of Márdia is 0.179. Critical ratios are statistically significant, which leads to the maintenance of all items. Figure 6a corresponds to the initial model, which excluded item Q6.7 due to multicollinearity problems, and shows that items Q6.1, Q6.4 and Q6.6 saturate below 0.40, leading to their elimination. The goodness of fit indices for the global adjustment in this initial model are only suitable for the GFI and SRMR, being poor for CFI and RMSEA and unsuitable for the chi-square ratio (Table 6). Figure 6b represents the final refined model without the eliminated items. It is observed that all items have saturations greater than 0.60, and individual reliability greater than 0.40. The goodness of fit indices for the overall adjustment are very good. As the results in Table 6 show, for the initial one-dimensional model only the chi-square ratio was inadequate, but in the final model, after refinement, the indices are excellent, saturated for all indices.

## 4. Discussion

The six dimensions of the EATMOT scale were all validated, thus confirming the adequacy of the selected types of reasons that could influence eating patterns and food choices: health and disease, emotional status, convenience and easiness of access, societal and cultural influences or religious beliefs, environmental concerns or political frameworks, and finally all issues related with marketing, advertising or promotional campaigns.

Regarding the health motivations scale, from the 10 initial questions considered, only 3 were validated, which reflects the fragility of the aspects considered. However, if we look in more detail to the questions validated (Q1.1: “I am very concerned about the hygiene and safety of the food I eat”, Q1.4: “It is important for me that my daily diet contains a lot of vitamins and minerals”, Q1.8: “It is important for me to eat food that keeps me healthy”), we see that they all focus on health as a general concern, indicating that the participants look upon health more as a general concept than as individual contributions or pathologies. This is expected, since a great deal of scientific evidence has been collected about the close relations between food intake and health status [31,32,33,34,35]. Another factor that may contribute to the importance attributed to some health issues is associated with gender differences. The sample under study was composed of far more women than men, and it has been reported that women tend to be generally more reflective about food and health issues [36,37].

In the original group of nine questions about emotional motivations, there were four items that remained after validation, and they are in fact those questions more directly related with anxiety and negative emotional mood, like stress and depression, which are in fact very strong emotions (Q2.1: “Food helps me cope with stress”, Q2.6: “When I feel lonely, I console myself by eating”, Q2.8: “For me, food serves as an emotional consolation, Q9.9: “I have more cravings for sweets when I am depressed”). Emotional eating is extremely powerful, and has been associated with the growing epidemic of obesity and related pathologies, owing to the propensity to balance negative emotions through food intake, particularly with unhealthy products such as those rich in sugar or fat [38]. Emotional eating is present among all age groups, although with a higher expression in younger individuals, and could even be considered a pathology that should be handled by mental health practitioners [39].

From the seven questions initially considered concerning convenience aspects that could influence food choices, three were retained, focusing on easiness of access to food, both in terms of facility to acquire and low price, and easiness of preparation (Q3.2: “The main reason for choosing a food is its low price”, Q3.3: “I choose the food I consume, because it is convenient to purchase”, Q3.7: “I prefer to buy food that is ready to eat or pre-cooked”). In today’s society, with “fast” life styles, where people seem to hardly find the time to do everything they wish to, the demand for convenience foods has grown, along with the distance between those who produce food and their consumers [37]. Many factors can justify this growing trend to seek convenience foods, like for example changes in the household structure, intensification of female employment, response of the food industry, marketing campaigns and advertisements, availability of kitchen technology compatible with cooked or pre-cooked meals, individualism, lack of time, or poor cooking skills [40,41,42,43,44].

The social and cultural motivations scale was initially composed of nine items, from which three were validated for the EATMOT scale (Q4.1: “Meals are a time of fellowship and pleasure”, Q4.2: “I eat more than usual when I have company”, Q4.8: “I like to try new foods to which I am not accustomed”). These focus on eating as a social act and the interaction between people while eating, as well as on the importance given to new foods and gastronomic experiences. According to Nakata [45], food tastes better and people tend to eat higher quantities when they are accompanied than when they are alone. Possible explanations can be assumed to justify this social facilitation of eating, some relying on the positive influence that company has on people by establishing social bounds, and others based on the assumption that people tend to imitate others, and therefore they eat because they see others eating. On the other hand, not only does company influence eating, but the type of social relationships is also considered an important factor for eating facilitation, so that when people are with friends or family they tend to ingest higher quantities of food than when they are in the presence of strangers [46,47,48].

Regarding environmental concerns and political motivations, from the initial seven items, the majority, four, were retained in the validated scale, which reflects the importance of aspects such as sustainability or human and animals’ rights when making food choices (Q5.1: “It is important to me that the food I eat is prepared/packed in an environmental friendly way”, Q5.3: “It is important to me that the food I eat comes from my own country”, Q5.4: “I prefer to eat food that has been produced in a way that animals’ rights have been respected”, Q5.5: “I choose foods that have been produced in countries where human rights are not violated”). Food is one of the three consumption domains with greater environmental impact, and therefore food consumption is a central aspect for a sustainable food supply [49,50]. Consumer inclination towards sustainable food purchase can help minimize food waste and residues, as well as packaging materials, and can also minimize the environmental impacts along the production chain, from the farm to the fork. Similarly, informed food choices can have a large impact on the well-being of farm animals [37]. Consumers may also benefit the local economy with their socially responsible choices [51]. However, we must bear in mind that sustainability should not jeopardize food choices in terms of nutritional values. One example is the ‘nutritional transition’ of dietary patterns, and the consumption of foods with higher content in animal protein, acknowledging that meat is considered as the food product with the greatest environmental impact throughout the food chain [52]. Grunert et al. [53] revealed in their research that sustainability labels still do not play a major role in consumers’ food choices. Finally, one of the latest studies on environmental modeling in the food chain revealed the following food related research perspectives: the environmental impacts of novel food processing technologies; innovative food packaging and changes in diets; and food consumption in connection with climate and environmental changes [54].

Finally, the last scale, concerning marketing issues, was initially composed of seven items, from which three were validated to be included in the EATMOT scale (Q6.2: “I eat what I eat, because I recognize it from advertisements or have seen it on TV”, Q6.3: “I usually buy food that spontaneously appeals to me”, Q6.5: “Food advertising campaigns increase my desire to eat certain foods”), and they confirm the important role of publicity and marketing as influencers on food choice. Food marketing has been identified as such an important determinant for obesity, that in many countries, restrictions have been imposed when advertising foods or beverages for children or adolescents. Further, the role of companies that sponsor many sports is believed to be influential in food consumption [55]. Care must be taken when it comes to advertisements. Although food marketing can be used to incentivize the consumption of healthy foods and beverages, the reality is sometimes different, and marketing is used to promote foods with a high energy density and low nutritional value. This is particularly true when it comes to young people, the demographic for whom food is one the most heavily marketed product categories. On the other hand, promotional campaigns can be an ally in eliciting the purchase of better quality foods at lower prices [56].

## 5. Conclusions

The results of this work allowed the assessing of six different types of factors that can influence food choices and eating behaviors, in the specific categories: health, emotions, price and availability, society and culture, environment and politics, and marketing and commercials. The results obtained for a wide sample from 16 countries indicated that all the individual scales measured presented good or very good fitting indices, with saturation in GFI in all cases, and values of chi-square ratio of 6.921 (for health), 0.987 (environment), 0.610 (emotions), and 0.000 in the remaining unifactorial models (convenience, society, marketing). Furthermore, the fit was perfect, with saturation for all indices, in three of the six models (convenience, society and marketing).

The scales validated include dimensions that are complementary and help in assessing food choice determinants, which can be very helpful in designing strategies that lead people to adopt better eating habits (those more oriented towards the maintenance of health, rather than having adverse effects). Furthermore, sustainability is a top priority in today’s society, and the food supply chain contributes a high impact on the biosystems, increasing water, soil and atmosphere pollution, which will in the end also impact people’s health. To understand people’s decision processes, and which factors drive them to make some choices instead of others, is pivotal in changing eating behaviors. These results produced six independent scales for assessing eating motivations, which can be applied in future situations with a high degree of confidence, bearing in mind that they were validated for a considerably vast sample, across different countries and with participants from different cultural backgrounds.

Some limitations of this study include the possible biases due to the unequal number of participants, like for example there being more female than male participants, or more participants from countries like Croatia or Portugal, factors that are associated with the use of convenience samples in different countries. These limitations are to some extent counterbalanced by the high number of participants overall.

## Figures and Tables

**Table 1:** Statistics for the unidimensional model of the scale about health motivations.

Item	Internal Consistency of Items (Original)				
	Mean Score	Standard Deviation	r (Item-Total)	r^2^	α Without Item
Q1.1	3.66	1.043	0.407	0.216	0.681
Q1.2	3.21	1.073	0.264	0.127	0.705
Q1.3	3.59	0.930	0.469	0.302	0.672
Q1.4	3.74	0.931	0.489	0.408	0.669
Q1.5	2.89	1.125	0.221	0.280	0.713
Q1.6	3.38	1.017	0.509	0.359	0.663
Q1.7	3.25	1.065	0.338	0.263	0.692
Q1.8	3.95	0.949	0.507	0.412	0.666
Q1.9	2.92	1.154	0.236	0.286	0.712
Q1.10	3.24	1.147	0.329	0.176	0.695
Global Cronbach’s alpha = 0.709					
**Fitting Indices of CFA Model ^1^**		**Initial ^2^**		**Final ^3^**	
χ^2^/df		228.0		6.921	
GFI		0.883		1.000	
CFI		0.668		0.999	
RMSEA		0.138		0.022	
RMSR		0.109		0.005	
SRMR		0.090		0.054	

^1^ χ^2^/df = Ratio of chi-square and degrees of freedom; GFI = Goodness of fit index; CFI = Comparative fit index; RMSEA = Root mean square error of approximation; RMSR = Root mean square residual; SRMR = Standardized root mean square residual. ^2^ Without modification indices. ^3^ With modification indices and eliminated items.

**Table 2:** Statistics for the unidimensional model of the scale about emotional motivations.

Item	Internal Consistency of Items (Original)				
	Mean Score	Standard Deviation	r (Item-Total)	r^2^	α Without Item
Q2.1	2.98	1.123	0.569	0.359	0.708
Q2.2	3.15	1.075	0.108	0.066	0.774
Q2.3	2.82	1.282	0.290	0.103	0.754
Q2.4	3.14	1.152	0.216	0.085	0.762
Q2.5	3.49	1.100	0.332	0.219	0.744
Q2.6	2.57	1.190	0.663	0.565	0.690
Q2.7	2.98	1.250	0.553	0.415	0.708
Q2.8	2.57	1.182	0.669	0.586	0.689
Q2.9	2.94	1.265	0.507	0.400	0.716
Global Cronbach’s alpha = 0.772					
**Fitting Indices of CFA Model ^1^**		**Initial ^2^**		**Final ^3^**	
χ^2^/df		105.0		0.610	
GFI		0.951		1.000	
CFI		0.901		1.000	
RMSEA		0.093		0.000	
RMSR		0.078		0.003	
SRMR		0.058		0.017	

^1^ χ^2^/df = Ratio of chi-square and degrees of freedom; GFI = Goodness of fit index; CFI = Comparative fit index; RMSEA = Root mean square error of approximation; RMSR = Root mean square residual; SRMR = Standardized root mean square residual. ^2^ Without modification indices. ^3^ With modification indices and eliminated items.

**Table 3:** Statistics for the unidimensional model of the scale about economic and availability motivations.

Item	Internal Consistency of Items (Original)				
	Mean Score	Standard Deviation	r (Item-Total)	r^2^	α Without Item
Q3.1	3.62	1.010	0.121	0.246	0.508
Q3.2	2.65	1.134	0.404	0.374	0.383
Q3.3	3.04	1.123	0.372	0.309	0.399
Q3.4	3.87	1.057	-0.072	0.166	0.582
Q3.5	3.17	1.091	0.327	0.155	0.422
Q3.6	2.78	1.079	0.411	0.226	0.384
Q3.7	2.41	1.188	0.164	0.342	0.497
Global Cronbach’s alpha = 0.500					
**Fitting Indices of CFA Model ^1^**		**Initial ^2^**		**Final ^3^**	
χ^2^/df		404.1		0.000	
GFI		0.867		1.000	
CFI		0.606		1.000	
RMSEA		0.184		0.000	
RMSR		0.141		0.000	
SRMR		0.124		0.000	

^1^ χ^2^/df = Ratio of chi-square and degrees of freedom; GFI = Goodness of fit index; CFI = Comparative fit index; RMSEA = Root mean square error of approximation; RMSR = Root mean square residual; SRMR = Standardized root mean square residual. ^2^ Without modification indices. ^3^ With modification indices and eliminated items.

**Table 4:** Statistics for the unidimensional model of the scale about social and cultural motivations.

Item	Internal Consistency of Items (Original)				
	Mean Score	Standard Deviation	r (Item-Total)	r^2^	α Without Item
Q4.1	3.74	1.034	0.178	0.219	0.487
Q4.2	3.06	1.107	0.338	0.179	0.433
Q4.3	2.65	1.072	0.273	0.133	0.457
Q4.4	2.44	1.090	0.359	0.194	0.426
Q4.5	2.61	1.131	-0.004	0.164	0.548
Q4.6	3.28	1.108	0.208	0.176	0.478
Q4.7	2.62	1.199	0.260	0.167	0.459
Q4.8	3.47	1.155	0.118	0.170	0.509
Q4.9	2.38	1.080	0.281	0.235	0.454
Global Cronbach’s alpha = 0.504					
**Fitting Indices of CFA Model ^1^**		**Initial ^2^**		**Final ^3^**	
χ^2^/df		321.7		0.000	
GFI		0.854		1.000	
CFI		0.348		1.000	
RMSEA		0.164		0.000	
RMSR		0.164		0.000	
SRMR		0.132		0.000	

^1^ χ^2^/df = Ratio of chi-square and degrees of freedom; GFI = Goodness of fit index; CFI = Comparative fit index; RMSEA = Root mean square error of approximation; RMSR = Root mean square residual; SRMR = Standardized root mean square residual. ^2^ Without modification indices. ^3^ With modification indices and eliminated items.

**Table 5:** Statistics for the unidimensional model of the scale about environmental and political motivations.

Item	Internal Consistency of Items (Original)				
	Mean Score	Standard Deviation	r (Item-Total)	r^2^	α Without Item
Q5.1	3.48	0.996	0.554	0.326	0.769
Q5.2	3.77	1.013	0.442	0.224	0.789
Q5.3	3.30	1.089	0.459	0.218	0.787
Q5.4	3.43	1.093	0.633	0.429	0.753
Q5.5	3.06	1.045	0.542	0.357	0.771
Q5.6	2.81	0.987	0.519	0.301	0.775
Q5.7	3.20	1.021	0.565	0.338	0.767
Global Cronbach’s alpha = 0.799					
**Fitting Indices of CFA Model ^1^**		**Initial ^2^**		**Final ^3^**	
χ^2^/df		93.44		0.987	
GFI		0.969		1.000	
CFI		0.937		1.000	
RMSEA		0.088		0.000	
RMSR		0.042		0.032	
SRMR		0.039		0.026	

^1^ χ^2^/df = Ratio of chi-square and degrees of freedom; GFI = Goodness of fit index; CFI = Comparative fit index; RMSEA = Root mean square error of approximation; RMSR = Root mean square residual; SRMR = Standardized root mean square residual. ^2^ Without modification indices. ^3^ With modification indices and eliminated items.

**Table 6:** Statistics for the unidimensional model of the scale about marketing and commercials motivations.

Item	Internal Consistency of Items (Original)				
	Mean Score	Standard Deviation	r (Item-Total)	r^2^	α Without Item
Q6.1	3.18	1.113	−0.049	0.121	0.498
Q6.2	2.30	1.013	0.352	0.301	0.257
Q6.3	2.67	1.130	0.274	0.290	0.299
Q6.4	3.69	1.067	−0.039	0.118	0.487
Q6.5	2.71	1.118	0.329	0.314	0.261
Q6.6	3.04	1.089	0.338	0.117	0.257
Q6.7	3.00	1.071	0.324	0.105	0.310
Global Cronbach’s alpha = 0.399					
**Fitting Indices of CFA Model ^1^**		**Initial ^2^**		**Final ^3^**	
χ^2^/df		163.8		0.000	
GFI		0.958		1.000	
CFI		0.854		1.000	
RMSEA		0.117		0.000	
RMSR		0.084		0.000	
SRMR		0.071		0.000	

^1^ χ^2^/df = Ratio of chi-square and degrees of freedom; GFI = Goodness of fit index; CFI = Comparative fit index; RMSEA = Root mean square error of approximation; RMSR = Root mean square residual; SRMR = Standardized root mean square residual. ^2^ Without modification indices. ^3^ With modification indices and eliminated items.

## Appendix A

The questions included in the questionnaire were distributed across six different sections as follows:

Section 1—Health motivations:
Q1.1. I am very concerned about the hygiene and safety of the food I eat;
Q1.2. It is important for me that my diet is low in fat;
Q1.3. Usually I follow a healthy and balanced diet;
Q1.4. It is important for me that my daily diet contains a lot of vitamins and minerals;
Q1.5. There are some foods that I consume regularly, even if they may raise my cholesterol;
Q1.6. I try to eat foods that do not contain additives;
Q1.7. I avoid eating processed foods, because of their lower nutritional quality;
Q1.8. It is important for me to eat food that keeps me healthy;
Q1.9. There are some foods that I consume regularly, even if they may raise my blood glycaemia;
Q1.10. I avoid foods with genetically modified organisms;
Section 2—Emotional motivations:
Q2.1. Food helps me cope with stress;
Q2.2. I usually eat food that helps me control my weight;
Q2.3. I often consume foods that keep me awake and alert (such as coffee, coke, energy drinks);
Q2.4. I often consume foods that help me relax (such as some teas, red wine);
Q2.5. Food makes me feel good;
Q2.6. When I feel lonely, I console myself by eating;
Q2.7. I eat more when I have nothing to do;
Q2.8. For me, food serves as an emotional consolation;
Q2.9. I have more cravings for sweets when I am depressed;
Section 3—Economic and Availability motivations:
Q3.1. I usually choose food that has a good quality/price ratio;
Q3.2. The main reason for choosing a food is its low price;
Q3.3. I choose the food I consume, because it is convenient to purchase;
Q3.4. I buy fresh vegetables to cook myself more often than frozen;
Q3.5. I usually buy food that is easy to prepare;
Q3.6. I usually buy food that it is on sale;
Q3.7. I prefer to buy food that is ready to eat or pre-cooked;
Section 4—Social and Cultural motivations:
Q4.1. Meals are a time of fellowship and pleasure;
Q4.2. I eat more than usual when I have company;
Q4.3. It is important to me that the food I eat is similar to the food I ate when I was a child;
Q4.4. I eat certain foods because other people (my colleagues, friends, family) also eat it;
Q4.5. I prefer to eat alone;
Q4.6. I choose the foods I eat, because it fits the season;
Q4.7. I eat certain foods because I am expected to eat them;
Q4.8. I like to try new foods to which I am not accustomed;
Q4.9. I usually eat food that is trendy;
Section 5—Environmental & Political motivations:
Q5.1. It is important to me that the food I eat is prepared/packed in an environmentally friendly way;
Q5.2. When I cook I have in mind the quantities to avoid food waste;
Q5.3. It is important to me that the food I eat comes from my own country;
Q5.4. I prefer to eat food that has been produced in a way that animals’ rights have been respected;
Q5.5. I choose foods that have been produced in countries where human rights are not violated;
Q5.6. I avoid going to restaurants that do not have a recovery policy of food surplus;
Q5.7. I prefer to buy foods that comply with policies of minimal usage of packaging;
Section 6—Marketing and Commercials motivations:
Q6.1. When I buy food I usually do not care about the marketing campaigns happening in the shop;
Q6.2. I eat what I eat, because I recognize it from advertisements or have seen it on TV;
Q6.3. I usually buy food that spontaneously appeals to me (e.g., situated at eye level, appealing colors, pleasant packaging);
Q6.4. When I go shopping I prefer to read food labels instead of believing in advertising campaigns;
Q6.5. Food advertising campaigns increase my desire to eat certain foods;
Q6.6. Brands are important to me when making food choices;
Q6.7. I try to schedule my shopping for when I know there are promotions or discounts.
